# Eco-Innovation and Innovation Level of the Economy as a Basis for the Typology of the EU Countries

**DOI:** 10.3390/ijerph19042005

**Published:** 2022-02-11

**Authors:** Elżbieta Sobczak, Dariusz Głuszczuk, Andrzej Raszkowski

**Affiliations:** Department of Regional Economy, Wroclaw University of Economics and Business, Nowowiejska 3, 58-500 Jelenia Góra, Poland; elzbieta.sobczak@ue.wroc.pl (E.S.); dariusz.gluszczuk@ue.wroc.pl (D.G.)

**Keywords:** eco-innovation, innovation, socio-economic development, typology of the European Union countries

## Abstract

The study addresses the issue of eco-innovation and innovation in the European Union countries, which is important from the perspective of the sustainable development paradigm. Innovation constitutes a significant factor related to socio-economic development, and it is crucial in constructing the competitive advantage of enterprises, regions, and countries. Nowadays, an increasing importance is attached to eco-innovations, which takes into account the ecological perspective ensuring the reduction of environmental burdens. The purpose of the conducted research was to assess the diversity among the European Union countries regarding the situation related to eco-innovation and innovation, which is focused on the typology of the EU Member States taking a holistic approach to innovation, i.e., considering not only economic but also environmental and social performance. The methods of multivariate statistical analysis, with a particular emphasis on classification methods, were used in the research. A holistic overview of innovation results from the combination of findings based on the research was carried out within the framework of the Eco-Innovation Observatory and the European Innovation Scoreboard. The study covered 28 European Union countries in the period 2013–2019. As a result of the conducted analyses, four classes of the EU Member States were identified (Leaders of Eco-Innovation and Innovation, Moderate Eco-Innovators and Catching-Up Leaders of Innovation, Poor Innovators, The Poorest Eco-Innovators and Innovators).

## 1. Introduction

When introducing the problem, it should be highlighted that the definitions of innovation proposed by the Oslo Manual are of an economic and business nature. For example, they specify business innovation as a new or improved product or a business process (or their combination), which is significantly different from the earlier business processes or products of the enterprise, which has been launched on the market or entered into operation by the enterprise. In other terms, innovation stands for the implementation of a new or significantly improved product (article or service) or process, a new marketing method, or a new operational method in business practice, workplace organization, or in relations with the environment [1].

Eco-innovation means the implementation of a new or significantly improved product (article or service), process, organizational change, or marketing solution that reduces using natural resources (including materials, energy, water, and land) and limits the release of harmful substances throughout the life cycle [2]. Innovativeness itself is a feature of economic entities or economies, meaning the ability to create and implement innovations, as well as their absorption, which is associated with active involvement in innovative processes and taking due actions in this respect; it also means involvement in acquiring the resources and skills indispensable to participate in these processes. Eco-innovation is part of the above understanding of innovation, with the difference that it also contributes to the reduction of burdens on the environment. The above-mentioned definitions and theoretical foundations include components of the Eco-Innovation Index and the Summary Innovation Index [3,4,5]; therefore, the following terms are used interchangeably in the study: situation in the field of innovation and eco-innovation—innovation and eco-innovation.

Certainly, the concept of eco-innovation is inextricably linked with sustainable development. For the purposes of an introduction to the research problems, the aforementioned development can be defined as the process of transformations that ensures that the needs of the present generation are met without diminishing the development opportunities of future generations, e.g., owing to integrated and comprehensive activities in the field of economic, social, and environmental development. The cited definition adopts that the economic and civilization development of the present generation should not take place at the expense of exhausting non-renewable resources and destroying the environment, for the benefit of future generations, who will also have the right to their development. This understanding of sustainable development was disseminated following the report publication by the World Commission on Environment and Development entitled Our Common Future. Further reinforcement of this idea was continued at the Earth Summit 1992 and resulted in publishing the document referred to as Agenda 21. The next key stage of the activities aimed at sustainable development was the United Nations Millennium Declaration, which defined the Millennium Development Goals. The implementation of these goals was focused on enhancing effective solutions to the 21st century problems by 2015. The provisions of the 1992 summit were renewed in 2002 in Johannesburg and later at the summit in Rio de Janeiro in 2012, which was referred to as Rio + 20. At the summit, the declaration The Future We Want was adopted, in which, among other things, the participants expressed their willingness to promote the idea of sustainable future on the economic, social, and environmental level. In 2015, the Millennium Development Goals were replaced by the Sustainable Development Goals (SDG) included in the Transforming Our World 2030 Development Agenda. The 2030 Agenda for Sustainable Development presents a development plan for the world, with the goal of eradicating poverty by 2030, a dignified life for all, and ensuring peace. Sustainable development, or rather the pursuit of its fullest achievement remains beyond any doubt one of the most important challenges faced by the modern world. It can be adopted that eco-innovation is one of the useful tools on the way toward the implementation of diversified SDGs [6,7,8,9,10,11,12,13,14,15,16,17,18,19,20,21,22,23,24,25,26,27,28,29,30,31].

Eco-innovations introduce new solutions in the field of environmental protection, with particular emphasis on the development toward green economy. Following this approach, the activities related to eco-innovation can be understood as an entrepreneurial procedure, covering the stage of product design and integrated management throughout its life cycle, which affects pro-ecological modernization of the economy by taking into account environmental problems and laws when developing products and the related processes. Eco-innovation can result in the reduction of material and energy inputs while increasing the quality of products or services. Eco-innovation basically refers to every sphere of enterprise operation, from technology, through products and services, to institutional and legal solutions. In terms of supporting eco-innovation-oriented activities, several key areas can be identified, such as e.g., using environmental policy and legislation to promote eco-innovation; supporting demonstration projects and establishing partnerships to introduce promising, intelligent, and forward-looking operational technologies to the market; developing new standards to stimulate eco-innovation; mobilization of financial instruments and support services for SMEs; promoting international cooperation in the field of eco-innovation; supporting the development of new skills and opening new jobs, as well as appropriate training programs aimed at adapting to the labor market needs taking into account environmental aspects; supporting eco-innovation through endogenous local and regional processes.

The implementation of eco-innovation is also supported by a high level of ecological education, also referred to as environmental education, which refers to the concept of upbringing, the subject of teaching as well as educational and upbringing-oriented activity, a system of shaping attitudes and views toward the surrounding world based on respect for the environment. Through a multifaceted and interdisciplinary approach, it makes people sensitive to environmental problems and threats, makes them aware of their causes and effects, teaches methods of solving them, including responsibility for the natural environment, and also remains an incentive for taking active actions, both as an individual and in a group aimed at protecting the natural environment.

Based on the above discussion, it can be adopted that eco-innovation stands for any innovation that results in the achievement of sustainable development through the reduction of negative impacts of manufacturing activities on the environment, increasing natural resilience to burdens or ensuring greater efficiency and responsibility in taking advantage of natural resources. The application of eco-innovations that favor the development of new processes, technologies, and services that make European businesses greener enhances the potential optimization for economic growth while addressing such challenges as climate change, scarcity of natural resources, and biodiversity loss. Eco-innovations also offer opportunities for enterprises. Their introduction contributes toward reducing the costs of running a business, allows taking advantage of new development opportunities, and has a positive impact on the company image. For this reason, the process of practical implementation of good ideas related to eco-innovation and industrial development should be accelerated through removing economic and legal barriers along with promoting investment, stimulating demand, and disseminating knowledge in the field of sustainable development [32,33,34,35,36,37,38,39,40,41,42,43,44,45,46,47,48,49,50,51,52,53,54,55,56,57,58,59,60,61,62,63].

Eco-innovation can also be defined as an intentional entrepreneurial behavior involving product design and integrated management throughout its life cycle that contributes to the ecological modernization of industrial age societies by considering environmental concerns when developing products and the related processes. Eco-innovation results in integrated solutions aimed at reducing resource and energy inputs while increasing product and service quality. Eco-innovation stands for any form of innovation that aims at making significant and demonstrable progress toward sustainable development by reducing environmental impact or achieving a more efficient and responsible use of natural resources, including energy. Eco-innovation is essentially the same as other types of innovation; however, it stands out in terms of two characteristics: eco-innovation represents innovation that leads to a reduction in environmental impact, whether or not such an effect is expected; the scope of eco-innovation can extend beyond the conventional organizational barriers of an innovative organization and cover a broader social involvement, which triggers changes within the existing socio-cultural standards and institutional structures. In other terms, eco-innovation means innovation in any form that results or aims at making significant and visible progress toward achieving sustainable development by reducing negative environmental impacts, increasing environmental resilience, or achieving a more efficient and responsible use of natural resources. Eco-innovations refer to the areas of business and technology in which the invention and implementation of modern products, manufacturing processes, services as well as management and promotional methods take place, the equivalent goal of which is to reduce environmental risk, pollution, or other negative impact on the natural environment, along with increasing profit and improving enterprise competitiveness; hence, all participants of economic life should take advantage of the benefits offered by eco-innovation [64,65,66,67,68,69,70,71].

The premise for undertaking empirical research was an attempt to answer the following research questions:

− Do any significant disproportions occur in the European Union countries regarding the level of eco-innovation and innovation of the economy in the spatial–temporal cross-section?
− Is there any convergence between the level of eco-innovation and innovation in the economies of the EU Member States?

In the presented study, the classification was based on a holistic approach to the innovation of economies, i.e., considering business and environmental aspects, while taking into account the Eco-Innovation Index and the Summary Innovation Index values, unlike in the reports of the European Union.

Innovation was measured using the Eco-Innovation Index and the Summary Innovation Index. The study covered 28 European Union countries in the years 2013–2019. The time scope of the research was based on the availability of comparable statistical data.

The purpose of the conducted research was both to assess the degree of diversity presented by the European Union countries in terms of the eco-innovation and innovation level and the division of the EU Member States into relatively homogeneous classes and also their typology, at the same time taking into account the situation in the field of eco-innovation and innovation.

## 2. Materials and Methods

### 2.1. Data

The classification of the European Union countries regarding their situation in terms of innovation and eco-innovation was based on two aggregate measures: the Eco-Innovation Index and the Summary Innovation Index.

The Eco-Innovation Index—developed by the Eco-Innovation Observatory (EIO is the initiative financed by the European Commission’s Directorate-General for the Environment)—aggregates 16 indicators systematized in five areas [3]:

- Eco-innovation inputs—indicators: (1) Governments environmental and energy R&D appropriations and outlays, (2) Total R&D personnel and researchers, (3) Total value of green early-stage investments—per capita;
- Eco-innovation activities—indicators: (4) Enterprises that introduced an innovation with environmental benefits obtained within the enterprise, (5) Enterprises that introduced an innovation with environmental benefits obtained by the end user, (6) ISO 14001 registered organizations;
- Eco-innovation outputs—indicators: (7) Eco-innovation-related patents, (8) Eco-innovation-related academic publications, (9) Eco-innovation-related media coverage),
- Resource efficiency outcomes indicators: (10) Material productivity, (11) Water productivity, (12) Energy productivity, (13) GHG emissions intensity;
- Socio-economic outcomes—indicators: (14) Exports of products from eco-industries, (15) Employment in eco-industries, (16) Turnover in eco-industries.

The Eco-Innovation Index illustrates the situation of the European Union Member States in terms of eco-innovation against the EU average (EU average = 100). In relation to the Summary Innovation Index, it is of complementary nature as it extends the innovation measurement by ecological aspects taking a holistic view of economic, environmental, and social results.

The Summary Innovation Index—in accordance with the European Innovation Scoreboard methodology—aggregates 27 indicators located in 10 dimensions of innovation [5]:

- Human resources—indicators: (1) New doctorate graduates, (2) Population aged 25–34 with tertiary education, (3) Lifelong learning;
- Attractive research systems—indicators: (4) International scientific co-publications, (5) Top 10% most cited publications, (6) Foreign doctorate students;
- Innovation-friendly environment—indicators: (7) Broadband penetration, (8) Opportunity-driven entrepreneurship;
- Finance and support—indicators: (9) R&D expenditure in the public sector, (10) Venture capital expenditures;
- Firm investments—indicators: (11) R&D expenditure in the business sector, (12) Non-R&D innovation expenditure, (13) Enterprises providing training to develop or upgrade ICT skills of their personnel;
- Innovators—indicators: (14) SMEs with product or process innovations, (15) SMEs with marketing or organizational innovations, (16) SMEs innovating in-house;
- Linkages—indicators: (17) Innovative SMEs collaborating with others, (18) Public–private co-publications, (19) Private co-funding of public R&D expenditure;
- Intellectual assets—indicators: (20) PCT patent applications, (21) Trademark applications, (22) Design applications;
- Employment impacts—indicators: (23) Employment in knowledge-intensive activities, (24) Employment fast-growing enterprises of innovative sectors;
- Sales impacts—indicators: (25) Medium and high-tech product exports, (26) Knowledge-intensive services exports, (27) Sales of new-to-market and new-to-firm product innovations.

The Summary Innovation Index is calculated as the arithmetic mean of the normalized values (0–1) of 27 indicators (comprehensive algorithm for determining the Summary Innovation Index—see [5]). For the purposes of this study, the European Innovation Scoreboard data were recalculated to determine the situation of individual European Union Member States in terms of innovation against the EU average in each year covered by the study (EU average = 100).

The spatial scope of the research covered all EU Member States. The spatial dimension of the analyses in relation to the possibility of obtaining data for 28 EU countries defined the time frame for the conducted research—the years 2013–2019. For example, Croatia was not included in the Eco-Innovation Observatory data until 2012 (EU Member State since 1 July 2013).

### 2.2. Statistical Analysis and Research Procedure

The methods of multivariate statistical analysis were used in conducting empirical research, with particular emphasis on Ward’s hierarchical classification methods and the k-means clustering method. More information about the classification methods can be found in the studies [72,73,74,75]. The calculations were performed using Statistica 13.1 package (Cracow, Poland). The research procedure was carried out in accordance with the following stages:

- Comparative analysis and diversity assessment of the European Union countries regarding the distribution of the Eco-Innovation Index and the Summary Innovation Index values in the years 2013–2019, using the basic descriptive parameters and their visualization in the form of box charts.
- Construction of Z block matrix of the normalized Eco-Innovation Index and the Summary Innovation Index values of the European Union countries taking the following form:

(1) $$Z=[Z^{EI}\vdots {Z^{I}]}_{\left(Nx2T\right)}=[z_{it}^{EI}\vdots {z_{it}^{I}]}_{\left(Nx2T\right)},\;$$

where $Z^{EI}\;$—matrix of the normalized Eco-Innovation Index values;

$Z^{I}$—matrix of the normalized Summary Innovation Index values;

i = 1, …, N—number of the analyzed object (country);

t = 1, …, T—number of the analyzed period,

(2) $$z_{it}^{S}=\frac{x_{it}^{S}-\underset{i}{\min}x_{it}^{S}}{R_{t}^{S}}$$

S∈{EI, I};

$z_{it}^{S}\;$—normalized value of S indicator for i-th object in t-th analyzed period;

$x_{it}^{S}\;$—value of S indicator for i-th object in t-th analyzed period;

$R_{t}^{S}\;$—range of S indicator values in t-th analyzed period.

The normalization of indicator values to the range [0, 1] was performed using the zero unitarization method based on the minimum value and range. The description of variable normalization and the applied properties are presented in the following studies: [76,77,78,79].

- Selecting the optimal number of classes of the studied countries based on the analysis of the dendrogram of connections, integration distances, and classification stages obtained using Ward’s hierarchical method. The choice of the number of classes (the cut-off point of the dendrogram) is made taking into account the distances between the successive nodes (they should be relatively large) and analyzing the agglomeration course graph. If there is a flattening on the agglomeration course graph, it means that at this point, the clusters are distant, which makes it a good cut-off point. The classification using Ward’s method was preceded by determining the squared Euclidean distance between the analyzed EU countries in terms of the studied indicators of eco-innovation and innovation.
- The division of EU countries into the relatively homogeneous classes using the k-means clustering method (the number of classes was determined in the previous stage of the analysis).
- Defining the typology and characteristics of the obtained classes covering the EU countries in terms of eco-innovation and innovation.

## 3. Results

**Figure 1:**
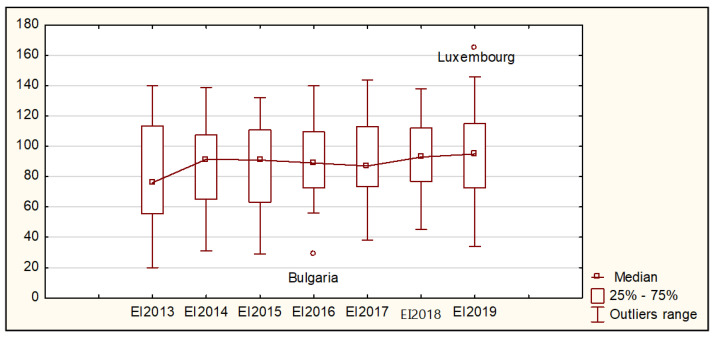
Box plot of the Eco-Innovation Index for the EU countries in the years 2013–2019. Source: authors’ compilation based on [3,4,5]. where: EI—Eco-Innovation Index.

The descriptive parameters defined separately for each period in the years 2013–2019 constituted the basis for assessing the diversity of European Union countries in terms of eco-innovation and innovation. The values of average measures, variation, and skewness are presented in Table 1 and Figure 1.

Figure 1 shows two outliers regarding the level of Eco-Innovation Index value. In 2016, Bulgaria recorded by far the lowest value of the Eco-Innovation Index, whereas Luxembourg showed the highest outlier value of the Eco-Innovation Index in 2019, as compared to other countries.

In the entire analyzed period, except for 2018, the lowest Eco-Innovation Index values were recorded in Bulgaria. In 2018 alone, the lowest level, i.e., 45% of the average Eco-Innovation Index value for the entire European Union, was observed in Cyprus. In 2016, Bulgaria was the outlier. The Eco-Innovation Index in this country reached only 29%, whereas in the other countries presenting the lowest ratings regarding eco-innovation, i.e., Cyprus and Poland, it amounted to 56%. Among the countries characterized with the highest eco-innovation, the following were listed: Luxembourg in 2014, 2016, and 2018–2019, Sweden in 2013 and 2017, and Germany in 2015. During the entire period under study, Luxembourg had the highest share in the EU average in relation to eco-innovation. In 2019, the Eco-Innovation Index in this country reached the value of 165%.

An increase in both the average value and the Eco-Innovation Index median was observed in the period under study. In 2019, on average, the Eco-Innovation Index was 94.07% of the EU average (87.08% in 2013). In 2019, in more than half of the EU countries, this indicator amounted to 95% of the EU average, while in 2013, it was only 76%. One-fourth of the countries (7) were characterized by the Eco-Innovation Index, which did not exceed 72.75% of the EU average, whereas in 2013, it was only 55.75%. In turn, the values of the third quartile did not change. In 2013, it was 113.25%, and in 2019, it was 113.5%, which means that across the analyzed years of the 14 EU countries, it did not exceed the level of approximately 113.5% of the analyzed indicator value. The diversity of the analyzed countries can be considered significant but decreasing (in 2013 CV = 44.44% and in 2019 CV = 34.60%). However, the variation measured by range increased from 120.00% in 2013 up to 131.00% in 2019. The distribution of the Eco-Innovation Index values showed variations both in terms of the direction and intensity of the asymmetry. In the last period of the study, the distribution of the Eco-Innovation Index values was almost symmetrical.

**Figure 2:**
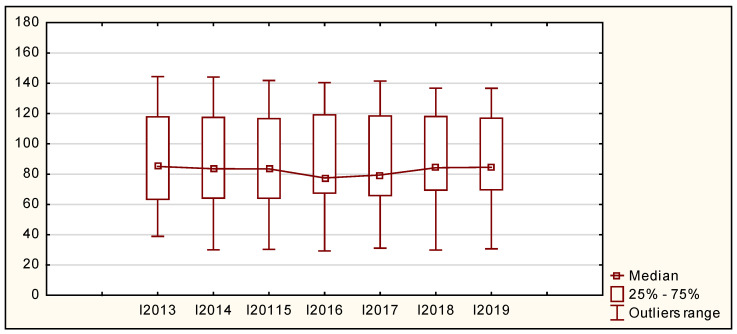
Box plot of the Summary Innovation Index for the EU countries in the years 2013–2019. Source: authors’ compilation based on [3,4,5]. where: I—Summary Innovation Index.

Table 2 and Figure 2 present the parameters characterizing the distribution of the Summary Innovation Index values in the European Union countries in the years 2013–2019.

In the entire analyzed period, Romania recorded the lowest Summary Innovation Index values in relation to the EU average, while in the first period of the study, this indicator amounted to 38.92%, and in the last period, it amounted to 30.71%. In the years 2013–2019, Sweden was the innovation leader in the EU. In 2013, its Summary Innovation Index was 144.35% of the EU average, and in 2019, it dropped to 136.69%. Both the average and the median of the Summary Innovation Index fluctuated slightly, remaining at a similar level at the beginning and end of the analyzed period, taking values of approximately 105% and 84%, respectively. In one-fourth of the EU countries, the value of the Summary Innovation Index did not exceed 63.35% in 2013 and 72.06% in 2019, which proves an improvement among the countries presenting the lowest innovation level comparing to the EU average. In turn, the value of the third quartile was at the level of approximately 117%, which means that the value of the Summary Innovation Index in 75% of the EU countries (21) did not exceed this level. The dispersion of the EU countries, measured by the coefficient of variation, was relatively large; however, it declined from 35.66% in 2013 to 31.52% in 2019. The range of this indicator outliers, along with some fluctuations, adopted the values of around 105% in the first and last of the analyzed years. In each year of the studied period, a positive asymmetry with its increasing intensity was observed. Bowley’s coefficient of skewness took positive values in the years 2013–2019. It means that in most EU countries, the Summary Innovation Index values, in each analyzed year, were lower than the average value. The highest intensity of asymmetry occurred in 2016 and 2017 when Bowley’s coefficient of skewness amounted to 0.62 and 0.50, respectively.

**Figure 3:**
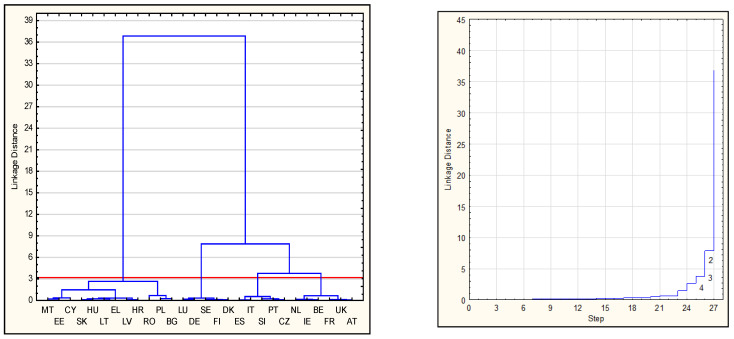
Dendrogram of connections, integration distances, and classification stages using Ward’s method for the EU countries. Source: authors’ compilation based on [3,4,5] using STATISTICA 13.1 statistical package. Note. Codes for EU countries: Austria (AT), Belgium (BE), Bulgaria (BG), Cyprus (CY), Czech Republic (CZ), Germany (DE), Denmark (DK), Estonia (EE), Greece (EL), Spain (ES), Finland (FI), France (FR), Croatia (HR), Hungary (HU), Ireland (IE), Italy (IT), Lithuania (LT), Luxembourg (LU), Latvia (LV), Malta (MT), Netherlands (NL), Poland (PL), Portugal (PT), Romania (RO), Sweden (SE), Slovenia (SI), Slovakia (SK), and the United Kingdom (UK). The red line indicates the cut-off level.

Figure 3 shows the dendrogram of connections, integration distances, and classification stages developed following the application of Ward’s hierarchical method, using squared Euclidean distance.

**Figure 4:**
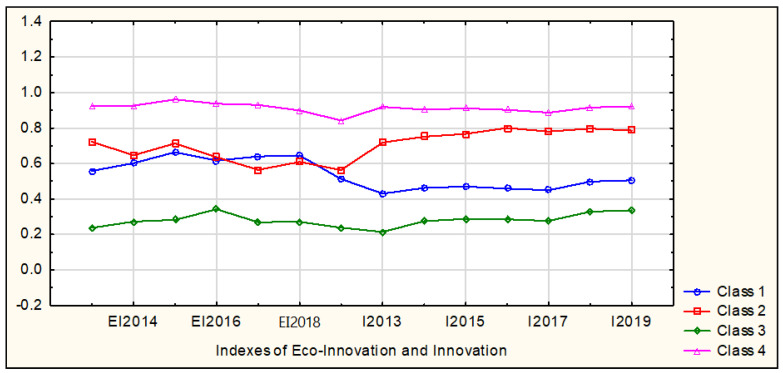
Mean values for the normalized indicators of the Eco-Innovation Index and the Summary Innovation Index of the EU countries in the years 2013–2019. Source: authors’ compilation based on [3,4,5] using STATISTICA 13.1 package. where: EI—Eco-Innovation Index, I—Summary Innovation Index.

Based on the analysis of the obtained results, the EU countries were divided into four relatively homogeneous classes using the k-means method. The final classification results are presented in Table 3 and Figure 4.

To determine whether there are statistically significant differences between four classes of the EU countries identified using the k-means method regarding the values of Eco-innovation and Innovation indices in 2013–2019, the non-parametric Kruskal–Willis test was used [80]. The statistics of the Kruskal–Wallis test are presented in Table 4.

Based on the test results presented in Table 4, it can be concluded that for the adopted significance level α = 0.05, the values of the eco-innovation and innovation indices in 2013–2019, in individual classes of the EU countries, showed significant differences. This also confirms that the number of classes was selected correctly.

The EU countries were divided into four relatively homogeneous classes regarding the values of the Eco-Innovation Index and the Summary Innovation Index (EU average = 100) in the years 2013–2019. These classes vary in size. The least numerous five-element classes represent the Leaders of Eco-Innovation and Innovation as well as Moderate Eco-Innovators and Poor Innovators. The most numerous is the Poorest Eco-Innovators and Innovators class, which includes 12 European Union countries. The third class (0.13) is characterized by the largest average distance from the middle of the class and thus also the highest diversity. The remaining classes of the EU countries show a similar distance from the middle of the class (from 0.07 to 0.08). In class 3, Bulgaria and Romania are the two countries most distant from the middle of the class, which are followed by Cyprus and Estonia. Table 5 and Table 6 present average values and coefficients of variation for the Eco-Innovation Index and the Summary Innovation Index, respectively, calculated for the EU countries in 2013–2019.

In the adopted assessment perspective, the countries classified in the fourth class (Sweden, Luxembourg, Germany, Finland, Denmark) were the leaders regarding the situation related to eco-innovation and innovation. In the years 2013–2019, this class recorded the average values of the Eco-Innovation Index and the Summary Innovation Index, respectively, ranging from 130.80 (2013) to 144.40 (2018) and from 127.80 (2018) to 135.83 (2013). The relatively high homogeneity in this class of countries is documented by a very low variation of the analyzed indicator values. In the years 2013–2019, the coefficient of variation (CV) of the Eco-Innovation Index ranged from 3.64% (2016) to 10.31% (2019), while in terms of the Summary Innovation Index, the values ranged from 5.64% (2013) to 7.02% (2017).

A polar opposite situation was recorded in the group of 12 Member States of the European Union (Bulgaria, Croatia, Cyprus, Estonia, Greece, Hungary, Latvia, Lithuania, Malta, Poland, Romania, Slovakia—third class). In this class, the average value of the Eco-Innovation Index in the years 2013–2019 ranged from 48.50 (2013) to 70.25 (2018), while the Summary Innovation Index ranged from 61.06 (2016) to 66.32 (2019). These results clearly differ from the values recorded within the remaining multi-element and relatively homogeneous groups of the EU countries, explicitly determining the peripherality of the third class. The absence of significant differences within this group is also worth noting, as evidenced by the average variation of the studied indicators. In 2013–2019, the coefficient of variation of the Eco-Innovation Index ranged from 21.09% (2018) to 30.60% (2013), and in the case of the Summary Innovation Index, it ranged from 23.98% (2017) to 27.58% (2019).

The countries included in the first (Czechia, Italy, Portugal, Slovenia, Spain) and the second (Austria, Belgium, France, Ireland, Netherlands, United Kingdom) classes share a relatively similar, moderate eco-innovation situation (Figure 4). In the years 2013–2019, their average Eco-Innovation Index values ranged from 87.00 (2013) to 105.80 (2017) in the first class and from 97.83 (2017) to 107.83 (2019) in the second class (Table 5). The significant differences between these groups resulted from the average Summary Innovation Index values, which was much lower in the first class (from 80.27 in 2016 to 84.30 in 2019) than in the second one (from 113.99 in 2019 to 118.26 in 2016). The range of average values in the Summary Innovation Index of the second class clearly indicates that this group of countries aspires to represent the innovation leaders, and their situation in this respect is not much worse than in the fourth group. It is worth adding that the analyzed indicators of eco-innovation and innovation within the first and second groups are characterized by very low variation.

## 4. Discussion

This study attempts to answer the following research questions:

− Do any significant disproportions occur in the European Union countries regarding the level of eco-innovation and innovation of the economy in spatial–temporal cross-section?
− Is there any convergence between the level of eco-innovation and innovation in the economies of the EU Member States?

In the years 2013–2019, the European Union countries showed significant differences both in terms of the Eco-Innovation Index and the Summary Innovation Index. In almost each year of the analyzed period, the standard deviation of the examined indicators exceeded 30% of their average values (CV > 30%). The year 2018 was the exception in this respect regarding the Eco-Innovation Index, when the value of the coefficient of variation was slightly lower (CV = 27.46%). Having analyzed the range of outliers, larger disproportions can be observed between the EU countries. The Summary Innovation Index value in Sweden, as the leader in the entire analyzed period, was more than four times higher than in Romania, which ranked as the last among the EU countries in terms of innovation (2013 is the exception—the indicator value was 3.71). A similar situation took place in the case of eco-innovation, although in 2013, in the extreme case, the value of Eco-Innovation Index in Sweden, being the leader, exceeded seven times the value of this indicator recorded for Bulgaria—the country ranked last in terms of eco-innovation. In the years 2013–2019, Luxembourg occupied the leading position four times regarding eco-innovation, Sweden twice, and Germany once. In turn, Bulgaria was ranked at the lowest position in the analyzed period (with the exception of 2018, when Cyprus came last in the EU). From this perspective, significant disproportions between the EU Member States should be acknowledged both in terms of the level of eco-innovation and innovation.

Throughout the analyzed period, the distribution of Summary Innovation Index values was characterized by a clearly positive asymmetry (see Table 2), which means that the countries featuring the average Summary Innovation Index value lower than the mean value prevailed. In the case of eco-innovation, such a phenomenon was not observed. The asymmetry of the distribution in the analyzed period showed a changing direction, and the asymmetry intensity was much lower.

When searching for the answer to the research question of whether the EU Member States showed similarities in terms of eco-innovation and innovation, they were divided taking into account the Eco-Innovation Index and the Summary Innovation Index values throughout the entire period under study. The empirical findings did not result in an explicit answer to this question. The situation in this respect was complicated and diversified. Namely, two classes of the EU countries were identified, in which the progress in the eco-innovation and innovation processes can be assessed in a similar manner. One of them is the fourth class, which is defined as the Leaders of Eco-Innovation and Innovation, which includes the countries developing eco-innovation and innovation in a convergent manner. In the analyzed period, the average Eco-Innovation Index and the Summary Innovation Index values in this group of countries presented the level of approximately 130% of the average value for all EU Member States. At the same time, it is noticeable that in the subsequent years of the 2013–2019 period, the average value of the Eco-Innovation Index was increasingly higher, thus strengthening the dominance of countries forming the leader class and achieving the average value of the Eco-Innovation Index in 2019 by 44% higher than the average value for the EU. On the other hand, in the case of innovation, the changes observed in this group went in the opposite direction. In 2013, in the countries forming the leader class, the average Summary Innovation Index value exceeded the EU average by almost 36%, and in 2019—the last year of the study, it exceeded the EU average by more than 28%. The fourth-class countries group the success factors. Their analysis by other EU Member States should be focused on searching for their own domestic opportunities to improve the situation in the field of eco-innovation and innovation. Moreover, it can be concluded that the fourth-class countries considered pro-ecological development, strengthening the environmental order, as their priority.

Another group of countries characterized by a similar level of eco-innovation and innovation is the third class, covering the Poorest Eco-Innovators and Innovators. The number of countries classified in this class, which can be considered peripheral in relation to others, raises particular concerns. It covers as many as 12 countries, including 11 countries from the so-called new enlargement of the EU (majority of them from the Central and Eastern Europe) and Greece as the only country from the so-called EU15 (Table 3). In this class of countries, the Eco-Innovation Index and the Summary Innovation Index values were the lowest in the studied timeframe oscillating, on average, around approximately 65% of their mean value for the EU. At the same time, in the analyzed period, the greater amplitude of changes was recorded in the case of eco-innovation (48% of the EU average in 2013 and 70.25% of the EU average in 2018. The countries included in the class covering the poorest eco-innovators and innovators should definitely focus their national economic policies on the development of innovation along with its pro-ecological orientation. As they belong to the less developed and less wealthy EU countries, therefore, the elimination of disproportions in terms of eco-innovation and innovation should be the main goal of the EU’s economic, environmental, and social policy. The poorest eco-innovators and innovators should take effective advantage of the EU pro-innovative financial instruments. The EU should increase the effectiveness of support aimed at the development of eco-innovation and innovation in the countries lagging in this respect.

In the other two classes of countries, i.e., Moderate Eco-Innovators and Catching-Up Leaders of Innovation (second class) and also Moderate Eco-Innovators and Poor Innovators (first class), as opposed to those previously characterized, different levels of eco-innovation and innovation were observed in the years 2013–2019. Both classes of countries are characterized by a similar moderate level of eco-innovation; however, in terms of innovation level, they should be differently assessed. In both classes, the average Eco-Innovation Index values present a similar level, taking slightly higher values in the second class—Moderate Eco-Innovators and Catching-Up Leaders of Innovation. In the analyzed period, the average Eco-Innovation Index values oscillate around the EU average, deviating by approximately 5% (the exception is 2013, when in the second class of the EU countries, the average Eco-Innovation Index value reached 87.00%, and in the first class, it reached 106.67%). The second class, Moderate Eco-Innovators and Catching-Up Leaders of Innovation, was characterized by the average Eco-Innovation Index values by several percent higher than the average value for the EU28 (approximately by 14% up to 18%). In turn, the first class—Moderate Eco-Innovators and Poor Innovators—showed the average Summary Innovation Index value by several percent lower than the average one for the EU28 (approximately by 15% to 19%).

As the above considerations show, high ranking in terms of the innovation situation is not always manifested in a definitely positive picture regarding eco-innovation. The second largest class consists of six countries, slightly deviating from the leaders in terms of innovation, but with worse, moderate results in the field of eco-innovation. This discrepancy seems to indicate, i.e., the need to develop and disseminate a holistic definition of innovation: the one taking into account not only the economic but also environmental effects (combining the definition of innovation with the idea of sustainable development and the need to protect the environment). On the other hand, a weak position in terms of innovation does not have to result in poor assessment regarding eco-innovation. The first class, similarly to the second class, is characterized by moderate results in the sphere of eco-innovation with an incomparable situation in terms of innovation. Thus, the innovation situation does not determine the eco-innovation performance and vice versa.

In a longer perspective, it is worth undertaking research addressing the identification of reasons for an even or uneven level of innovation and eco-innovation in the European Union countries, with particular emphasis on the factors enhancing and strengthening the capacity of economies toward developing and implementing eco-innovations and innovations, as well as barriers in this respect among the active and passive eco-innovative or innovative entities. It should also be considered necessary to classify and search for the countries—leaders in eco-innovation and innovation, along with determining factors of their success. Taking into account the natural environment and the objective need for sustainable development, it seems justified to conduct a combined, rather than separate, research on eco-innovation and innovation. The indicated research directions may come across limitations related to the availability of comparable data provided by the official statistics, which persistently collect statistical information distinguishing between eco-innovation and innovation. As a consequence, the research barriers emerge, which narrow down the scope of possible analyses.

## 5. Conclusions

The following conclusions can be formulated based on the conducted research and statistical analyses as well as the previously presented source literature studies:

- The EU countries show a clear divergence in terms of both eco-innovation and innovation. Moreover, significant disproportions were observed between the Eco-Innovation Index and the Summary Innovation Index outliers (over fourfold differences between the leading country and the country ranked as last);
- The EU countries taking the leading positions in terms of eco-innovation are simultaneously the leaders in relation to innovation. This group includes Sweden, Luxembourg, Germany, Finland, and Denmark. These countries should constitute a benchmark for other EU Member States and be subject to further specific monitoring carried out by researchers;
- The EU countries belonging to the group of the poorest eco-innovators are at the same time the poorest in terms of innovation. It is the most numerous class grouping 11 countries from the so-called new EU enlargement, also including Greece;
- Among the countries characterized by a moderate position in terms of eco-innovation, there are both countries catching up with the leaders regarding innovation and the ones presenting a poor position in terms of innovation;
- Eco-innovations are closely related to the methods of using natural resources as well as the production and consumption processes, and also to the concepts of eco-efficiency and green industry. Eco-innovation fosters the shift of manufacturing enterprises from the “end-of-pipe” technology to “closed-loop” solutions that minimize material and energy flows by changing products and production methods, thus resulting in a competitive advantage of many enterprises and sectors;
- A clean and healthy environment is very important for maintaining prosperity and high quality of life in Europe. In order to ensure it, a competitive economy taking advantage of eco-innovative solutions is needed;
- Designing and promoting new eco-innovative solutions is needed to use the potential for achieving economic benefits through cost savings, innovation, and international trade;
- Environmentally friendly projects can attract new types of high-tech manufacturing and services, increase the competitiveness of the European Union, and create highly qualified job opportunities;
- We are currently facing major environmental challenges such as climate change, depletion of natural resources, and biodiversity loss. New economic and social models as well as technologies resulting in explicit and significant environmental benefits are indispensable. These models can make extensive use of eco-innovative solutions;
- Eco-innovations can help European entrepreneurs develop sustainable solutions which make better use of valuable resources and reduce the negative impacts of the economy on the environment. From this perspective, eco-innovations remain a helpful tool based on which we can manage the existing resources more efficiently and contribute to green economic growth.

Based on the conducted research, it is possible to formulate several recommendations supporting the development policies implemented by the commercial entities and the public sector. Eco-innovation depends on the overall level of innovation, which should be comprehensively supported. In other words, effective pro-innovation policies represent an indispensable condition for increasing the eco-innovation of the entire economy. Part of the innovation support instruments should be adapted to the specificity of eco-innovation, including, e.g., technology certification. The information policy addressed to enterprises is also important. Companies have to perceive the opportunities related to eco-innovation in their specific case and understand the respective legal conditions. In addition, it is important to promote consulting services toward the green economy and environmental management systems. Effective actions performed by public administration are essential. It is crucial to provide an example and support the creation of new markets (e.g., green public procurement) as well as enforce the compliance with environmental regulations by enterprises. It is also necessary to coordinate the activities of individual public institutions responsible for eco-innovation. Appropriate funding is recommended, as well as the elimination of financial barriers. It refers, in particular, to limiting the harmful effects of economic activity on the environment. It should be borne in mind that eco-innovation largely depends on the appropriate support infrastructure, including the potential of modern IT solutions.

## Figures and Tables

**Table 1:** Descriptive parameters of the Eco-Innovation Index for the European Union countries in the years 2013–2019 (EU average = 100).

Statistics	2013	2014	2015	2016	2017	2018	2019
min	20.00 BG	31.00 BG	29.00 BG	29.00 BG	38.00 BG	45.00 CY	34.00 BG
max	140.00 SE	139.00 LU	132.00 DE	140.00 LU	144.00 SE	138.00 LU	165.00 LU
R	120.00	108.00	103.00	111.00	106.00	93.00	131.00
Md	76.00	91.50	91.00	89.00	87.00	93.00	95.00
$\bar{x}$	80.78	87.48	86.15	90.37	92.33	93.00	94.07
Q1	55.75	65.00	64.00	75.25	73.75	79.00	72.75
Q3	113.25	105.75	110.00	109.25	113.00	112.00	113.50
CV	44.44	33.40	33.99	30.41	31.51	27.46	34.60
SB	0.30	−0.30	−0.17	0.19	0.32	0.15	−0.09

Where: BG—Bulgaria, CY—Cyprus, SE—Sweden, LU—Luxembourg, DE—Germany, R—Range; Md—Median; $\bar{x}$—Arithmetic Mean; Q1, Q3—first and third Quartiles; CV—Coefficient of Variation (%); SB—Bowley’s Coefficient of Skewness. Source: authors’ own calculations based on [3,4,5].

**Table 2:** Descriptive parameters of the Summary Innovation Index value for the European Union countries in the years 2013–2019 (EU average = 100).

Statistics	2013	2014	2015	2016	2017	2018	2019
min	38.92 RO	29.99 RO	30.23 RO	29.29 RO	31.14 RO	29.90 RO	30.71 RO
max	144.35 SE	144.10 SE	141.81 SE	140.45 SE	141.39 SE	136.83 SE	136.69 SE
R	105.44	114.10	111.58	111.17	110.26	106.92	105.98
Md	85.08	83.54	83.45	77.46	79.44	84.23	84.53
$\bar{x}$	90.25	89.66	89.86	88.99	88.96	90.24	90.84
Q1	63.35	64.31	64.23	67.74	66.59	71.95	72.06
Q3	117.71	116.94	116.42	118.89	117.95	117.91	116.72
CV	35.66	35.52	34.64	35.04	34.28	32.16	31.52
SB	0.20	0.27	0.26	0.62	0.50	0.47	0.44

Where: RO—Romania, SE—Sweden, R—Range; Md—Median; $\bar{x}$—Arithmetic Mean; Q1, Q3—first and third Quartiles; CV—Coefficient of Variation (%); SB—Bowley’s Coefficient of Skewness. Source: authors’ calculations based on [3,4,5].

**Table 3:** Classification of the European Union countries based on the Eco-Innovation Index value and the Summary Innovation Index value in the years 2013–2019 (EU average = 100).

No.	Typology of Classes	Class Composition	Class Size	Average Distance from the Middle of the Class
1.	Moderate Eco-Innovators and Poor Innovators	Czechia, Italy, Portugal, Slovenia, Spain	5	0.08
2.	Moderate Eco-Innovators and Catching-Up Leaders of Innovation	Austria, Belgium, France, Ireland, Netherlands, United Kingdom	6	0.07
3.	The Poorest Eco-Innovators and Innovators	Bulgaria, Croatia, Cyprus, Estonia, Greece, Hungary, Latvia, Lithuania, Malta, Poland, Romania, Slovakia	12	0.13
4.	Leaders of Eco-Innovation and Innovation	Sweden, Luxembourg, Germany, Finland, Denmark	5	0.07

Source: Authors’ compilation.

**Table 4:** Kruskal–Wallis test statistics for four classes of the European Union countries identified using the k-means method regarding the values of the Eco-Innovation Index and Summary Innovation Index in 2013–2019.

Eco-Innovation Index			Summary Innovation Index		
Index	H	*p*	Index	H	*p*
EI2013	22.53	0.0001	I2013	22.59	0.0000
EI2014	22.60	0.0000	I2014	22.08	0.0001
EI2015	23.28	0.0000	I2015	22.08	0.0001
EI2016	21.83	0.0001	I2016	22.48	0.0001
EI2017	22.48	0.0001	I2017	22.34	0.0001
EI2018	22.08	0.0001	I2018	21.85	0.0001
EI2019	22.68	0.0000	I2019	21.68	0.0001

Where: EI—Eco-Innovation Index, I—Summary Innovation Index, H—Kruskal–Wallis test value, *p*—the lowest probability at which the null hypothesis can be rejected. Source: authors’ calculations based on [3,4,5].

**Table 5:** Descriptive parameters of the Eco-Innovation Index value for the selected types of classes of the European Union countries in the years 2013–2019 (EU average = 100).

No.	Class	Parameters	Years						
			2013	2014	2015	2016	2017	2018	2019
1.	Moderate Eco-Innovators and Poor Innovators	$\bar{x}$	87.00	96.00	97.40	97.40	105.80	105.00	101.20
		CV	25.20	10.55	9.20	11.33	13.22	4.62	7.07
2.	Moderate Eco-Innovators and Catching-Up Leaders of Innovation	$\bar{x}$	106.67	100.83	102.50	99.50	97.83	101.67	107.83
		CV	12.49	7.33	9.37	11.87	11.20	13.75	14.60
3	The Poorest Eco-Innovators and Innovators	$\bar{x}$	48.50	60.50	58.08	67.00	66.58	70.25	65.25
		CV	30.60	25.19	22.34	23.60	21.56	21.09	22.06
4.	Leaders of Eco-Innovation and Innovation	$\bar{x}$	130.80	131.00	128.00	133.00	136.60	128.60	144.40
		CV	7.90	5.18	3.75	3.64	6.96	7.90	10.31

Where: $\bar{x}$—Arithmetic Mean; CV—Coefficient of Variation (%). Source: authors’ compilation based on [3,4,5].

**Table 6:** Descriptive parameters of the Summary Innovation Index value for the selected types of classes of the European Union countries in the years 2013–2019 (EU average = 100).

No.	Class	Parameters	Years						
			2013	2014	2015	2016	2017	2018	2019
1.	Moderate Eco-Innovators and Poor Innovators	$\bar{x}$	84.26	82.76	82.72	80.27	80.74	83.15	84.30
		CV	11.67	11.83	11.97	10.27	8.42	6.27	6.50
2.	Moderate Eco-Innovators and Catching-Up Leaders of Innovation	$\bar{x}$	114.64	115.74	115.83	118.26	117.14	115.11	113.99
		CV	6.55	5.38	5.30	4.76	4.67	6.06	6.93
3	The Poorest Eco-Innovators and Innovators	$\bar{x}$	61.55	61.39	62.35	61.06	61.64	65.11	66.32
		CV	27.01	27.42	26.95	24.35	23.98	27.51	27.58
4.	Leaders of Eco-Innovation and Innovation	$\bar{x}$	135.83	133.08	131.86	129.64	128.91	127.80	128.43
		CV	5.64	7.01	6.33	6.22	7.02	6.47	6.83

Where: $\bar{x}$—Arithmetic Mean; CV—Coefficient of Variation (%). Source: authors’ compilation based on [3,4,5].

## Data Availability

European innovation scoreboard 2021—Database https://ec.europa.eu/docsroom/documents/46934, accessed on 22 December 2021. The eco-innovation scoreboard and the eco-innovation index The Eco-Innovation Scoreboard and the Eco-Innovation Index | Eco-innovation Action Plan (europa.eu).
